# Vasoactive Intestinal Peptide-Producing Neuroblastic Tumors: A Rare Cause of Refractory Diarrhea

**DOI:** 10.7759/cureus.67861

**Published:** 2024-08-26

**Authors:** Luiza Dornelles Penteado Pacheco e Silva, Eliana M Monteiro Caran

**Affiliations:** 1 Pediatric Oncology, Grupo de Apoio ao Adolescente e a Criança com Câncer (GRAACC) / Universidade Federal de São Paulo (UNIFESP), Sao Paulo, BRA

**Keywords:** vipoma, vasoactive intestinal peptide, ganglioneuroma, ganglioneuroblastoma, neuroblastoma, paraneoplastic syndrome, diarrhea

## Abstract

Neuroblastic tumors are the most common malignant extracranial solid tumors of childhood. A small subgroup presents chronic incoercible diarrhea due to the tumor’s production of vasoactive intestinal peptide (VIP). The hypothesis of an occult tumor is not always considered, which delays and impairs treatment. We aim to identify these patients’ characteristics and help alert health professionals to the hypothesis of a neuroblastic tumor in children with chronic diarrhea refractory to the usual approach. We carried out an epidemiological study on all retrievable reports of neuroblastic tumors between 1975 and 2021 described in the Medical Literature Analysis and Retrieval System Online (MEDLINE), Excerpta Medica database (EMBASE), and Latin American & Caribbean Health Sciences Literature (LILACS) databases. Patient information was divided into categories, and we performed a descriptive analysis. We analyzed 96 cases; 83 (86.5%) cases had diarrhea prior to the diagnosis of the neoplasm, 49 (51%) were ganglioneuroblastomas, 69 (71.8%) were abdominal, and 59 of the 60 patients (98%) with reported acid-base disorders had hypokalemia. When serum VIP was reported, the majority of values varied between one and 20 times the upper reference limit. Seventy-two (75%) patients underwent complete tumor resection, and the overall survival rate was 70%. Serum VIP production by neuroblastic tumors is related to cell differentiation and better prognosis. Such children often require intensive hospital support to reverse the malnutrition and acid-base disorders related to this paraneoplastic syndrome. Its early diagnosis and treatment significantly change the prognosis and quality of life. We, therefore, suggest screening for neuroblastic tumors when health professionals encounter unmanageable chronic secretory diarrhea in children with no defined etiology in the usual investigations.

## Introduction and background

In Brazil, the estimated annual incidence of childhood and young adult cancer (0-19 years) is 134.81 new cases per one million inhabitants [1]. Neuroblastic tumors are the most frequent among extracranial malignant solid neoplasms and account for 8%-10% of malignant tumors in this age group [2]. Neuroblastic tumors originate from a migrating cell subpopulation derived from the neural crest during embryonic life. They can occur anywhere along the sympathetic trunk from the cervical region to the pelvis, including the paravertebral sympathetic ganglia and the medullary region of the adrenals. The retroperitoneum (adrenal gland) is the most frequently affected site. In 90% of cases, it is possible to measure the production of catecholamines in urine and blood and use such levels for diagnosis and follow-up. Neuroblastic tumors range from differentiated, benign neoplasms (ganglioneuroma), differentiated mixed tumors with areas of immature cells (ganglioneuroblastoma), and tumors formed by undifferentiated malignant cells (neuroblastoma (NB)) [3]. Neuroblastoma is the most common and is responsible for 12% to 15% of childhood cancer deaths [4].

The average age of children with NB ranges from 0 to four years (median 19 months), and it is sporadic after the age of 10 [2]. The clinical presentation of NB is very heterogeneous and depends on primary tumor location, systemic symptoms, presence of metastases, and paraneoplastic syndromes [5]. Occasionally, patients are asymptomatic (13%) and the tumor is diagnosed on incidental findings of chest X-rays or ultrasounds ordered for other causes or after tumor palpation in a routine abdominal examination. Distension, pain, and constipation can occur in the abdomen depending on tumor size. Most patients (46%) present with aggressive and metastatic disease at diagnosis, and the most common sites are bone, liver, lymph nodes, and bone marrow infiltration [4,5]. 

The heterogeneity of the clinical presentation and the similarity of the signs and symptoms to other childhood diseases present a challenge to early diagnosis. The occurrence of paraneoplastic syndromes of occult primary tumors also poses a challenge for early diagnosis. The main paraneoplastic syndromes associated with neuroblastic tumors are opsoclonus-myoclonus syndrome, which occurs in 3% of cases, and diarrhea caused by vasoactive intestinal peptide (VIP) secretion, which occurs in approximately 1% of patients [6, 7]. Literature suggests that VIP production usually derives from more differentiated neural crest tumors, such as ganglioneuromas and ganglioneuroblastomas [7]. 

Despite its rarity, diarrhea secondary to VIP secretion by neuroblastic tumors is severe due to its brutal clinical repercussions and difficulties in diagnosis and treatment. Most times, the child is taken to the pediatrician with a sudden onset of profuse, watery diarrhea several times a day [8, 9]. The general condition is usually good, but the child does not improve despite hydration measures and may develop hydroelectrolytic and acid-base disorders such as hypokalemia, hyponatremia, metabolic acidosis, dehydration, and even malnutrition [10, 11]. The hypothesis of an occult neuroblastic tumor is not always considered, which delays or impairs treatment, which consists of surgery, chemotherapy, the use of somatostatin analogs, or somatostatin itself [12, 13]. Cases are sporadic, with no known association between familiar cancer syndromes, such as multiple endocrine neoplasia (MEN) syndromes, and VIP-producing neuroblastic tumors. Literature reports a neuroblastic tumor diagnosed by autopsy [14]. We carried out this literature review to assist in educating healthcare providers about the possibility of a neuroblastic tumor in children with chronic diarrhea refractory to the standard approach.

## Review

Methods

We reviewed the Medical Literature Analysis and Retrieval System Online (MEDLINE), Excerpta Medica database (EMBASE), and Latin American & Caribbean Health Sciences Literature (LILACS) databases, including all case reports of patients with NB, ganglioneuroblastoma, or ganglioneuroma who had chronic diarrhea between 1975 and 2021, which contained available full text.

**Figure 1:**
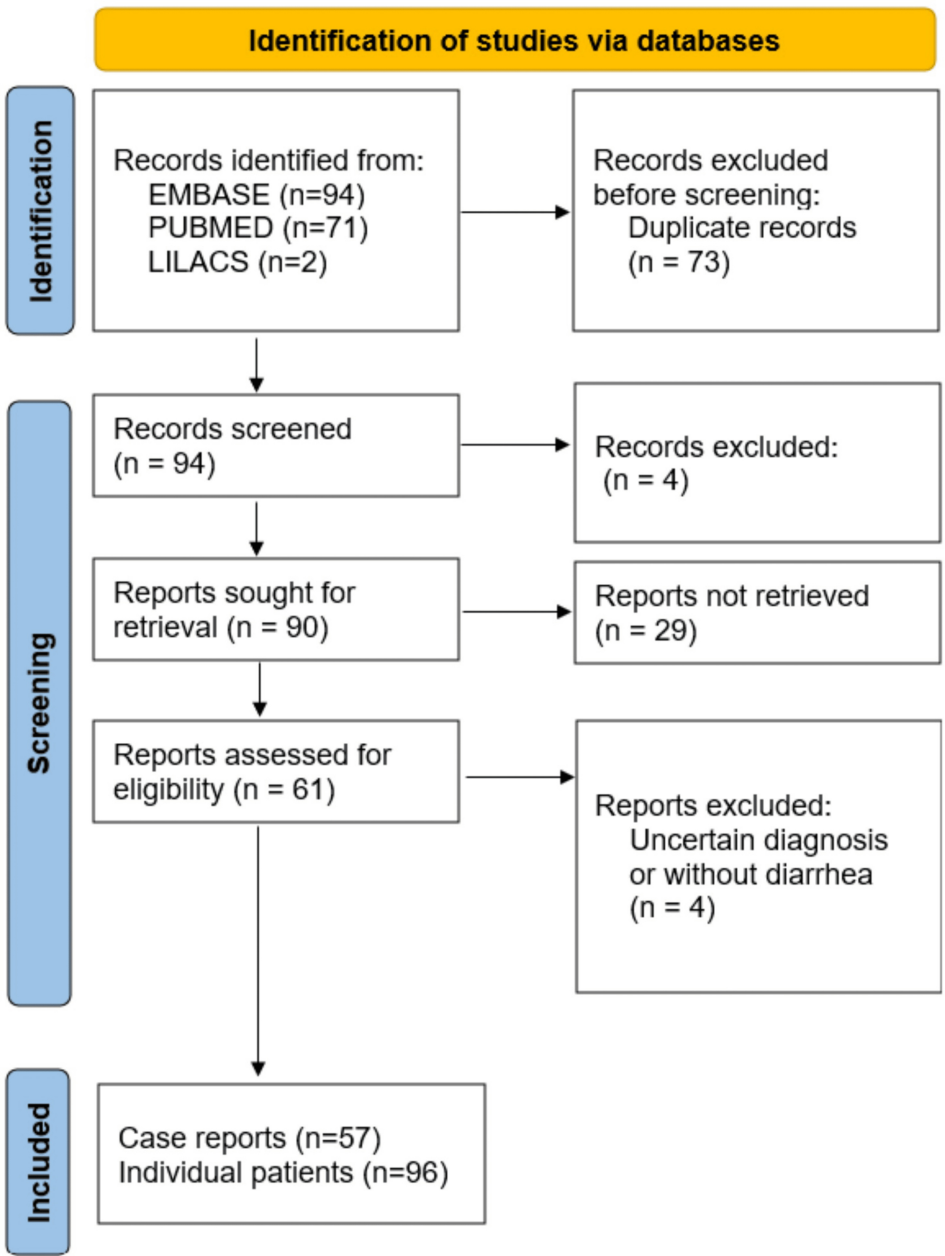
Article selection diagram based on PRISMA 2020 guidelines Adapted from Page MJ et al., 2020 [15] PRISMA: Preferred Reporting Items for Systematic Reviews and Meta-Analyses; MEDLINE: Medical Literature Analysis and Retrieval System Online; EMBASE: Excerpta Medica database; LILACS: Latin American & Caribbean Health Sciences Literature

We used the following keywords in the MEDLINE, EMBASE, and LILACS databases: "Neuroblastoma", "Ganglioneuroblastoma", "Ganglioneuroma", "Neural Crest tumor", "paravertebral mass", "mediastinal mass", "neurogenic tumor" and "tumor", joined by combined Boolean terms and the keywords "diarrhea", "paraneoplastic syndrome", "weight loss", "primary syndrome", "secondary syndrome", "Vasoactive Intestinal Peptide", "neuronal peptide", "vipoma", "verner-morrison syndrome", "VIP-secreting tumor", "WDHA", "WDHH", "WDHA syndrome", "WDHH syndrome". We also linked pediatric keywords already established in the literature. We found 167 articles compatible with the scope of the research, 73 of which were duplicates; therefore, 94 were valid articles or single case reports. Only 61 articles had retrievable full texts. Three of the articles were in Russian, Chinese, and German and their texts were translated into English using an automatic translator for this project. Due to uncertain pathological diagnosis or absence of diarrhea, four articles and 13 patients were excluded from the case series. Thus, we selected 57 case reports totaling 96 patients from 1975 to 2021. A summary is illustrated in Figure 1 [15].

Patient information was then classified into the following categories: year, author, age at diagnosis, age at onset of symptoms, gender, type of tumor, location of primary tumor, metastases, acid-base disorders, hydroelectrolyte disorders, MYCN expression, serum VIP level, treatment performed, and outcome, whenever the information was available. Serum VIP levels were then converted to times over the upper limit normal (ULN) reported by the authors for comparison. We performed a descriptive analysis of this population.

Results

Individual information from each patient is in Table 1 [7-14,16-62].

**Table 1:** Clinical, epidemiological, treatment, and survival data for patients with neuroblastic tumors and paraneoplastic diarrheal syndrome from 1975 to 2021 F: female; GN: ganglioneuroma; GNB: ganglioneuroblastomas; HypoCa: hypocalcemia; HypoC: hypochloremia; HypoK: hypokalemia; HypoNa: hyponatremia; HypoP: hypophosphatemia; M: male; N: no; N/A: not available in the article; NB: neuroblastoma; O: octreotide; PR: partial resection; QT: chemotherapy; RT: radiotherapy; RTot: total resection; ULN: upper limit normal; VIP: vasoactive intestinal peptide; Som: somatostatin; Y: yes

N	Author, year	Age at diagnosis (months)	Age of onset of symptoms (months)	Sex	Type of tumor	Location of the tumor	Metastases	Hydroelectrolytic/ acid-base disorder	VIP serum level (xULN)	MYCN amplified?	Treatment	Outcome
1	Swift et al., 1975 [8]	60	30	F	GN	Mediastinum	N	N/A	5.84	N/A	PR	Remission after recurrence
2	Mitchell et. al., 1976 [16]	17	12	M	GNB	Paravertebral	N	HypoK	2.25	N/A	RTot	Remission
3	Jansen-Goemans et. al., 1977 [17]	17	13	M	GNB	Cervical	N	HypoK	12.9	N/A	RTot	Remission
4	Collin et. al., 1979 [18]	8	11	M	GNB	Cervical	N	HypoK	1.44	N/A	RTot	Remission
5	Hansen et al, 1980 [19]	30	13	F	GN	Retroperitoneal	Y	HypoK	4.25	N/A	RTot	Remission
6	Iida et al., 1980 [20]	8	7	M	GNB	Adrenal	N	HypoK	3.04	N/A	RTot	Remission
7	Kaplan et al., 1980 [21]	24	21	M	GN	Paravertebral	N	HypoK + metabolic acidosis	7.5	N/A	RTot	Remission
8	Laburthe et al., 1980 [22]	35	32	F	GNB	Mediastinal	N	HypoK	5	N/A	N/A	N/A
9	Tiedemann et al., 1981 [12]	18	15	F	GNB	Adrenal	Y	HypoK + metabolic acidosis	14.4	N/A	PR + RT + QT + Som	Death due to disease
10	Cooney et. al., 1982 [23]	7	11	F	NB	Retroperitoneal	Y	HypoK + metabolic acidosis	7.87	N/A	RTot 2x	Remission after recurrence
11	Funato et al., 1982 [24]	23	17	F	GNB	Paravertebral	N	HypoK	32.5	N/A	RTot + QT + RT	Remission
12	Kudo et al., 1982 [25]	47	21	F	GNB	Adrenal	Y	HypoK	49	N/A	Surgery without tumor resection	Death due to disease
13	Scheibel et al., 1982 [26]	18	16	F	GNB	Mediastinal and Retroperitoneal	N	N/A	5	N/A	RTot + QT + RT	Remission
14	Yagihashi et al., 1982 [27]	36	N/A	F	GNB	Adrenal	Y	HypoK	49	N/A	Surgery for pancreatoduodenectomy, without tumor identification or resection	Death due to disease
15	Yamashiro et al., 1982 [28]	23	21	F	GN	Adrenal	N	HypoK, HypoCl	5.1	N/A	RTot	N/A
16	Booth et al., 1983 [29]	36	N/A	N/A	GNB	Adrenal	N	HypoK + metabolic acidosis	5.46	N/A	RTot	Remission
17	El Shafie et al., 1983 [9]	24	19	F	GNB	Adrenal	N	HypoK	19.02	N/A	RTot	Remission
18	El Shafie et al., 1983 [9]	12	11	M	GNB	Retroperitoneal	Y?	HypoK	51.38	N/A	PR + RT + QT	Remission
19	Granot et al., 1983 [30]	84	48	M	GN	Adrenal	N	N/A	3.2	N/A	RTot	Remission
20	Bunnett et al., 1984 [31]	12	N/A	F	GNB	Retroperitoneal	N	N/A	N/A	N/A	RTot	Remission
21	Schuman et al., 1984 [32]	8	3	M	GN	Adrenal	N	HypoNa	2.37	N/A	RTot	Remission
22	Socha et al., 1984 [14]	84	8	M	GN	Adrenal	N	HypoK	6.73	N/A	N	Death due to disease
23	Socha et al., 1984 [14]	36	16	M	GN	Adrenal	N	HypoK	3.13	N/A	RTot	Remission
24	Dorney et al., 1984 [33]	12	9	F	GN	Paravertebral	N	HypoK, HypoNa, HypoCl, HypoP	2.5	N/A	RTot	Remission
25	Oberg et al., 1986 [13]	5	N/A	M	NB	Retroperitoneal	Y	HypoK + metabolic acidosis	18	N/A	PR + Som	N/A
26	Quak et al., 1988 [34]	36	33	F	GN	Paravertebral	N	HypoK	10.4	N/A	RTot	Remission
27	Lacey et al., 1989 [35]	30	N/A	F	GNB	Mediastinal	N	HypoK	4.76	N/A	PR (2x) + RT + QT	Remission
28	Davies et al., 1990 [36]	14	10	M	GNB	Adrenal	N	HypoK	9.2	N/A	RTot	Remission
29	Kimura et al., 1993 [37]	18	N/A	F	GNB	Retroperitoneal	N	HypoK	7.6	N/A	RTot	N/A
30	Kimura et al., 1993 [37]	13	N/A	M	GNB	Retroperitoneal	Y	HypoK	3.18	N/A	RTot	N/A
31	Kimura et al., 1993 [37]	27,6	N/A	M	GNB	Retroperitoneal	N	HypoK	2.25	N/A	RTot	N/A
32	Albers et al., 1998 [38]	48	30	F	GNB	Adrenal	N	HypoK, HypoNa, HypoCa + metabolic acidosis	31.32	N/A	RTot	N/A
33	Murphy et al., 2000 [39]	28	10	M	GN	Paravertebral	N	N/A	N/A	N/A	RTot	Remission (follow-up ranging from 5-14 years)
34	Murphy et al., 2000 [39]	16	10	M	GN	Presacral	N	HypoK	4.6	N/A	RTot	Remission (follow-up ranging from 5-14 years)
35	Murphy et al., 2000 [39]	26	10	M	GN	Paravertebral	N	HypoK, HypoNa + metabolic acidosis	4.8	N/A	RTot	Remission (follow-up ranging from 5-14 years)
36	Murphy et al., 2000 [39]	13	7	F	GN	Adrenal	N	HypoK	5.1	N/A	RTot	Remission (follow-up ranging from 5-14 years)
37	Murphy et al., 2000 [39]	24	20	M	GNB	Retroperitoneal	N	N/A	N/A	N/A	RTot	Remission (follow-up ranging from 5-14 years)
38	Murphy et al., 2000 [39]	9	7	M	GNB	Adrenal	N	HypoK	4.3	N/A	RTot	Remission after recurrence (follow-up ranging from 5-14 years)
39	Rodriguez et al., 2000 [40]	22	17	F	GNB	Adrenal	N	HypoK + metabolic acidosis	11.8	N	RTot	Remission
40	Riebel et al., 2002 [41]	14	N/A	M	GNB	Retroperitoneal	N	HypoK, HypoNa + metabolic acidosis	15	N/A	RTot	Remission
41	Wildhaber et al., 2003 [42]	19	15	F	GNB	Presacral	N	HypoK	38.4	N	RTot + QT	Remission
42	Bourgois B et. al., 2004 [43]	14	13	F	NB	Mediastinum	Y	HypoK + metabolic acidosis	21,6	N	RTot	Remission
43	Gesundheit et al., 2004 [44]	27	19	F	GNB	Adrenal	N	N/A	N/A	N	RTot	N/A
44	Reindl et al., 2004 [45]	13	13	M	GNB	Paravertebral	N	HypoK, HypoNa + metabolic acidosis	37.5	N/A	RTot	N/A
45	Reindl et al., 2004 [45]	14	12	M	GNB	Paravertebral	N	N/A	0.47	N/A	RTot	N/A
46	Zhang et al., 2008 [46]	36	30	F	GN	Paravertebral	N	HypoK, HypoNa	33.2	N/A	RTot	Remission
47	Bourdeaut et al., 2009 [7]	25	15	N/A	GN	Adrenal	N	N/A	11	N/A	RTot	Remission
48	Bourdeaut et al., 2009 [7]	18	11	N/A	NB	Adrenal	N	N/A	8.3	N	RTot	Remission
49	Bourdeaut et al., 2009 [7]	44	23	N/A	GNB	Adrenal	N	N/A	11.1	N/A	RTot	Remission
50	Bourdeaut et al., 2009 [7]	18	16	N/A	GNB	Chest	N	N/A	6.5	N	RTot	Remission
51	Bourdeaut et al., 2009 [7]	38	28	N/A	GNB	Adrenal	N	N/A	2.7	N	RTot	Stable disease
52	Bourdeaut et al., 2009 [7]	19	14	N/A	NB	Abdominal	N	N/A	8.9	N	RTot	Remission
53	Bourdeaut et al., 2009 [7]	14	12	N/A	GNB	Chest	N	N/A	20.7	N	RTot	Remission
54	Bourdeaut et al., 2009 [7]	23	14	N/A	GNB	Abdominal	N	N/A	5	N/A	RTot	Death from other causes
55	Bourdeaut et al., 2009 [7]	13	3	N/A	GNB	Adrenal	N	N/A	11.1	N	RTot	Remission
56	Bourdeaut et al., 2009 [7]	9	7	N/A	NB	Adrenal	N	N/A	4.5	Y	RTot	Remission
57	Bourdeaut et al., 2009 [7]	11	8	N/A	NB	Abdominal	N	N/A	3.17	N	RTot	Remission
58	Bourdeaut et al., 2009 [7]	15	14	N/A	NB	Abdominal	N	N/A	4.9	N	RTot	Remission
59	Bourdeaut et al., 2009 [7]	17	14	N/A	GNB	Adrenal	Y	N/A	2	N	RTot	Stable disease
60	Bourdeaut et al., 2009 [7]	46	47	N/A	GNB	Abdominal	N	N/A	17.6	N/A	RTot	Death due to disease
61	Bourdeaut et al., 2009 [7]	27	28	N/A	NB	Adrenal	N	N/A	6.7	N/A	RTot	Stable disease
62	Bourdeaut et al., 2009 [7]	16	17	N/A	GNB	Adrenal	N	N/A	2.4	Y	RTot	Stable disease
63	Bourdeaut et al., 2009 [7]	33	38	N/A	NB	Adrenal	N	N/A	2.3	N	RTot	Progressive disease
64	Bourdeaut et al., 2009 [7]	17	18	N/A	NB	Adrenal	N	N/A	1.1	Y	RTot	Stable disease
65	Bourdeaut et al., 2009 [7]	27	36	N/A	GNB	Abdominal	N	N/A	1.9	Y	RTot	Progressive disease.
66	LeLeiko et al., 2010 [47]	18	12	F	GN	Paravertebral	N	N/A	1.8	N/A	RTot	Remission
67	Husain et al., 2011 [10]	18	16	F	GNB	Mediastinum	N	HypoK, HypoNa, HypoCl + metabolic acidosis	2.92	N/A	RTot + Som	N/A
68	Kargl et al., 2013 [48]	14	8	F	GNB	Paravertebral	N	HypoK	11.32	N/A	RTot	Remission
69	Kanik et al., 2014 [11]	15	11	F	GNB	Adrenal	N	HypoK + metabolic acidosis	3.92	N/A	RTot	Remission
70	Toro, Gonzales et al., 2014 [49]	20	12	M	GNB	Pre-sacral	Y	HypoK	99.6 pmol/L	N/A	RTot	Remission
71	Kumar et al., 2015 [50]	14	N/A	M	GNB	Paravertebral	Y	HypoK, HypoNa + metabolic acidosis	3.04	N/A	QT	N/A
72	Minson et al., 2015 [51]	11	13	F	GNB	Pelvic	N	HypoK	20	N	PR + QT	Death due to disease
73	Whitfield et al., 2015 [52]	24	16	M	NB	Pelvic	N	HypoK + metabolic acidosis	>1	N	QT + O	N/A
74	Romanyshyn et al., 2017 [53]	24	16	M	GN	Adrenal	N	HypoK	N/A	N/A	RTot	Remission
75	Sintusek et al., 2017 [54]	17	N/A	M	NB	Adrenal	N	HypoK + metabolic acidosis	N/A	N	RTot + QT	Remission
76	Tanzi et al., 2017 [55]	26	16	F	GNB	Mediastinum	N	Mild HypoK	6.86	N/A	RTot + QT	Remission
77	Czkwianianc et al., 2018 [56]	17	12	F	GNB	Retroperitoneal	N	HypoK, HypoNa	222 pmol/L	Y	RTot + QT	Remission
78	Czkwianianc et al., 2018 [56]	24	17	F	GNB	Retroperitoneal	Y	N/A	73 pmol/L	N	RTot + QT	Remission
79	Kabalan et al., 2018 [57]	11	9	F	NB	Retroperitoneal	N	HypoK, HypoNa + metabolic acidosis	N/A	N	PR + QT	Stable disease
80	Sugita et al., 2020 [58]	10	6	F	NB	Adrenal	N	HypoK	N/A	N	RTot + O	N/A
81	Uskova et al., 2020 [59]	10	N/A	F	NB	Retroperitoneal	Y	HypoK, HypoNa + metabolic acidosis	N/A	N	RTot + QT	Remission
82	Uskova et al., 2020 [59]	22	17	F	NB	Mediastinum and retroperitoneum	Y	HypoK, HypoNa + metabolic acidosis	N/A	N	2xPR + QT	Stable disease
83	Uskova et al., 2020 [59]	22	16	F	NB	Adrenal	N	HypoK + metabolic acidosis	N/A	N	RT	Remission
84	Uskova et al., 2020 [59]	7	6	M	NB	Retroperitoneal	Y	HypoK, HypoNa + metabolic acidosis	N/A	N	PR + QT	Death due to disease
85	Uskova et al., 2020 [59]	12	N/A	F	NB	Retroperitoneal	Y	HypoK + metabolic acidosis	N/A	N	RTot + QT	In treatment
86	Uskova et al., 2020 [59]	15	12	M	NB	Retroperitoneal	Y	HypoK + metabolic acidosis	N/A	N/A	QT	In treatment
87	Yeh P et al., 2020 [60]	11	10	M	GNB	Adrenal	Y	HypoK, HypoNa, HypoCl, HypoP, HypoCa	2.7	N/A	PR	N/A
88	Liu D et al., 2021 [61]	35	N/A	F	NB	Retroperitoneal	N	HypoK	N/A	N	PR + QT + O	Remission
89	Liu D et al., 2021 [61]	30	N/A	M	GNB	Adrenal	N	Not specified	N/A	N	PR + QT + O	Remission
90	Shahid et al., 2021 [62]	17	N/A	N/A	NB	Abdominal	N	N/A	1.21	N/A	RTot + QT	N/A
91	Shahid et al., 2021 [62]	21	N/A	N/A	NB	Abdominal	Y	N/A	1	N/A	QT	N/A
92	Shahid et al., 2021 [62]	21	N/A	N/A	NB	Abdominal	N	N/A	0.92	N/A	QT	N/A
93	Shahid et al., 2021 [62]	24	N/A	N/A	NB	Abdominal	Y	N/A	0.35	N/A	QT	N/A
94	Shahid et al., 2021 [62]	24	N/A	N/A	NB	Abdominal	Y	N/A	0.46	N/A	QT	N/A
95	Shahid et al., 2021 [62]	19	N/A	F	NB	Abdominal	Y	N/A	>8	Y	RTot + QT + O	Death due to disease
96	Shahid et al., 2021 [62]	11	N/A	F	NB	Abdominal	Y	N/A	3.54	Y	RTot + QT + O	Remission

The age at diagnosis of neuroblastic tumors ranged from five to 84 months; the mean was 23.5 months. As for gender, out of 96 patients, 40 were female and 25 had no information. The sex ratio was 1.3 females to one male.

There were three reported subtypes of neural crest tumors: 18 (18%) ganglioneuromas, 29 (30%) NBs, and 49 (51%) ganglioneuroblastomas. The MYCN oncogene was reported in seven of 96 (7.2%) patients, three of which were ganglioneuroblastomas and four NBs. The most common primary site of tumors was the abdominal cavity, which was divided into a grouping of 36 adrenal tumors, 17 retroperitoneal tumors, and 16 tumors not specifically identified in the articles, named only "abdominal," totaling 69 (71.8%) of 96 tumors. Out of 96, the most prevalent sites were 11 (11.4%) paravertebral cases and eight (8.3%) mediastinal cases. In the remaining categories, we identified two (2%) cases each: cervical, pelvic, pre-sacral, and thoracoabdominal.

Concerning the onset of diarrhea, 83/96 (86.5%) cases presented the symptom before oncologic diagnosis, and 13/96 (13.5%) cases presented post-diagnosis. The time interval between onset of diarrhea and tumor diagnosis was only reported in 64 of the 96 patients and ranged from 0 to 76 months, with a mean of 8.03 months.

**Figure 2:**
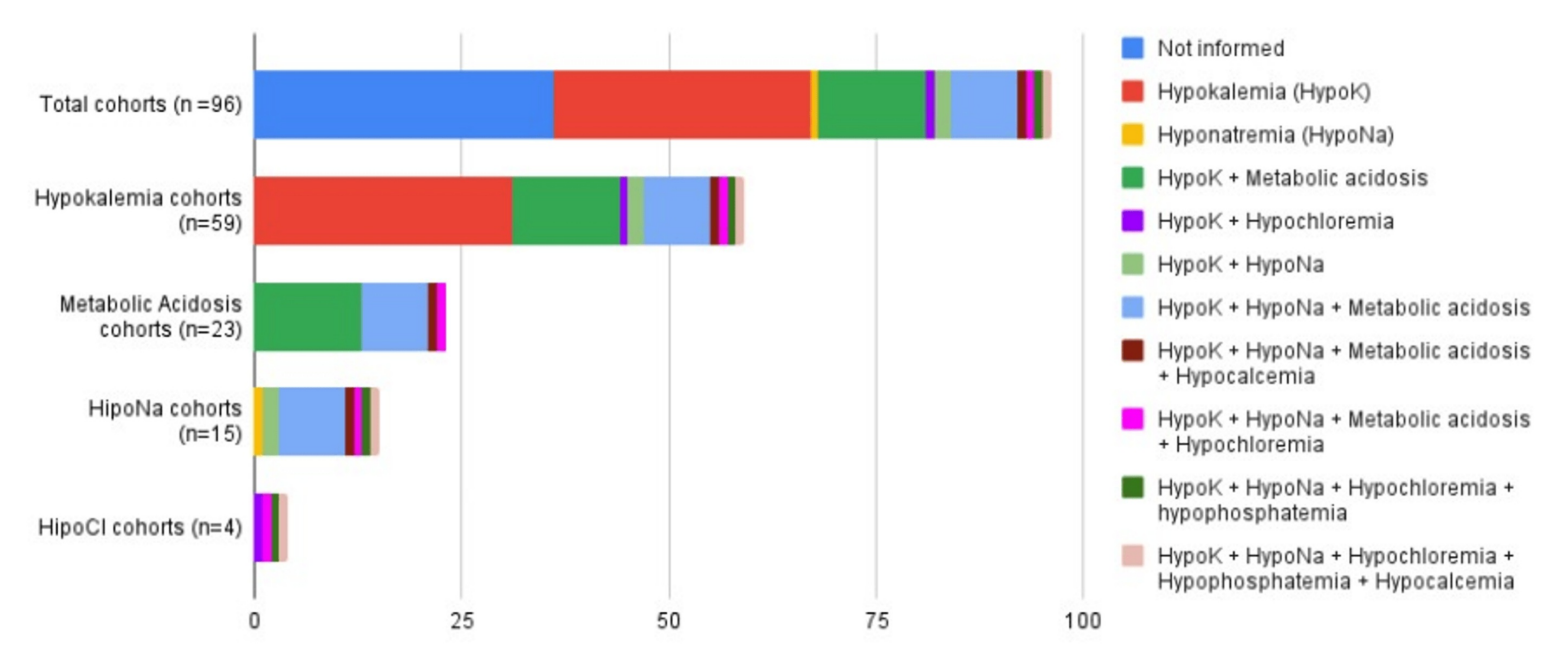
Hydroelectrolytic and acid-base disorders in vasoactive intestinal peptide-producing neural crest tumor patients This image has been created by the authors.

Out of 96 patients, hydroelectrolytic or acid-base disorders were described in 60 (62%) patients. Of these 60 patients, 59 (98.3%) had hypokalemia and the only patient without hypokalemia had isolated hyponatremia (1.7%). Hypokalemia was associated with metabolic acidosis in 23 (38.9%) of 59 cases and hyponatremia in 14 (23.7%) cases. Among the 59 participants, 10 (16.9%) cases had associated hypokalemia, hyponatremia, and metabolic acidosis. There were also two (3.3%) hypocalcemia cases, associated with hypokalemia and hyponatremia; one of these two cases (1.6%) was also hypophosphatemic. Four (6.7%) out of 59 cases had hypochloremia associated with hypokalemia, and one (1.6%) of these four cases had hypochloremia and hypokalemia associated with hyponatremia, with no association with metabolic acidosis. These data are illustrated in Figure 2. 

**Figure 3:**
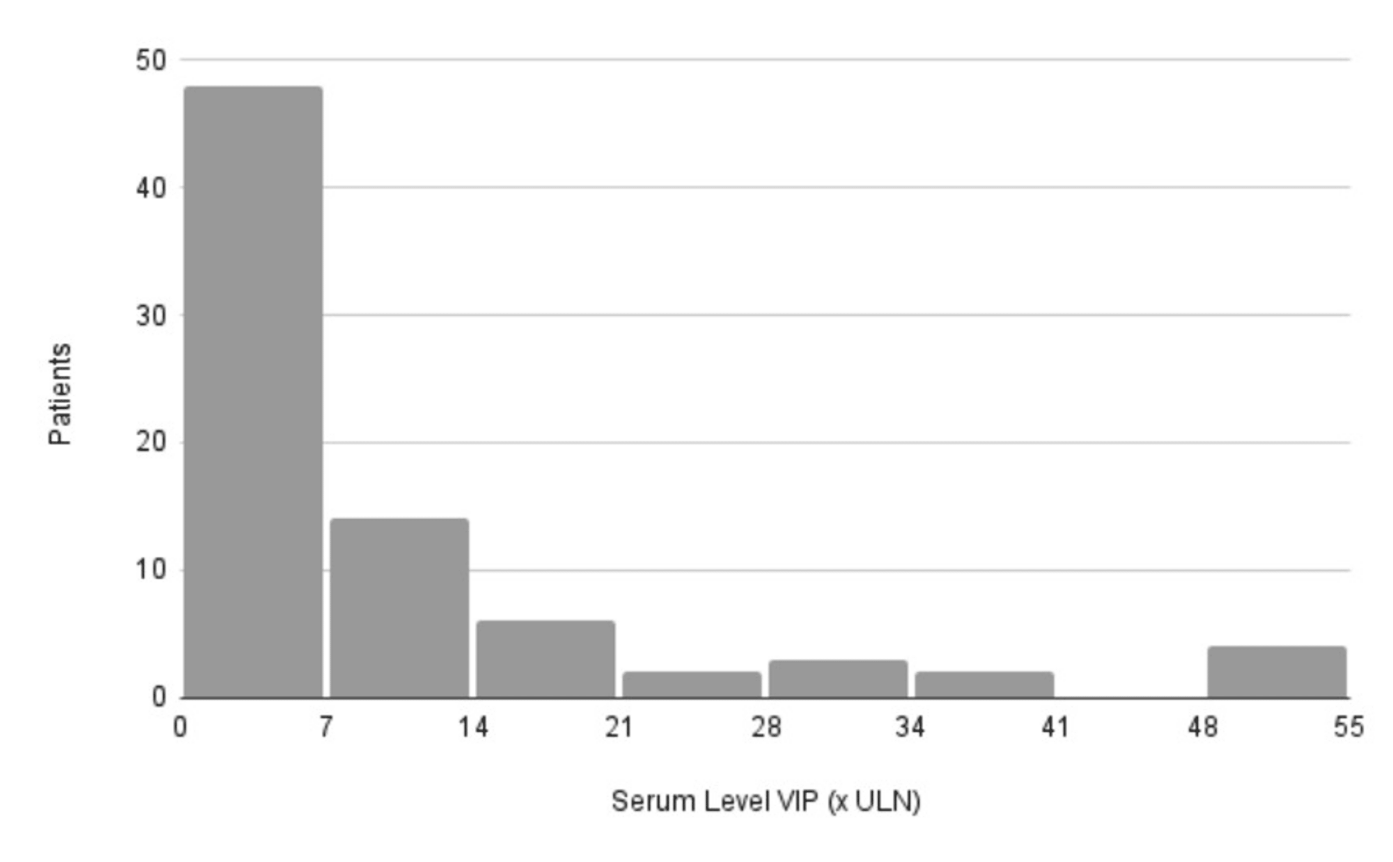
Comparison of vasoactive intestinal peptide serum levels VIP: vasoactive intestinal peptide; ULN: upper limit normal

Serum VIP levels were compared using the ULN reference values reported by the authors. The lowest VIP normal value was registered by El Shafie (1983) [9] (6-36 pg/ml), and the highest VIP normal value was registered by Tanzi (2017) [55] (<75 mg/ml). Seventy-seven of 96 (80%) patients had serum VIP measurements described, and two patients were excluded from the analysis because the test's reference value was missing [49, 56]. Because its value was very different from the rest of the sample (188 x ULN), in one patient we compared the measurement before chemotherapy began (14.4 x ULN) [12]. The value ranged from less than one to 51.38 x ULN. The mean was 13.9 x ULN. This comparison is illustrated in Figure 3.

Various treatment modalities were identified: surgery, with partial or total resection, chemotherapy, radiotherapy, and the use of somatostatin analogs or somatostatin itself. Among 96 patients, 84 (87.5%) underwent resection, of which 72 (85%) were complete and 12 (15%) were partial. A total of 25 (26%) of 96 patients received chemotherapy, 18 (72%) of which were associated with total surgery, six (24%) were not operated on and received concomitant radiotherapy instead, and three (12%) were given concomitant octreotide. Additionally, out of 96 patients, 11 (11.4%) were given somatostatin analogs, seven (7.2%) were given octreotide, and four (4.1%) were given somatostatin, with five (5.2%) also undergoing resection. Full treatment data can be found in Table 2.

**Table 2:** Treatment and survival data for patients with neuroblastic tumors and paraneoplastic diarrheal syndrome from 1975 to 2021 N: no; N/A: not available in the article; O: octreotide; PR: partial resection; QT: chemotherapy; R: radiotherapy; TR: total resection; Som: somatostatin; Y: yes

Author, year	N	Surgery	Type of resection	Use of somatostatin analogs	Other therapy	Status: alive	Survival
Swift et al., 1975 [8]	1	Y	PR	N	N	Y	2 weeks
Mitchell et. al., 1976 [16]	1	Y	TR	N	N	Y	11 months
Jansen-Goemans et. al., 1977 [17]	1	Y	TR	N	N	Y	12 months
Collin et. al., 1979 [18]	1	Y	TR	N	N	Y	12 months
Hansen et al, 1980 [19]	1	Y	TR	N	N	Y	12 months
Iida et al., 1980 [20]	1	Y	TR	N	N	Y	16 months
Kaplan et al., 1980 [21]	1	Y	TR	N	N	Y	4 months
Laburthe et al., 1980 [22]	1	Y	TR	N	N	N/A	N/A
Tiedemann et al., 1981 [12]	1	Y	PR	Y	QT and R	N	-
Cooney et. al., 1982 [23]	1	Y (2x)	TR	N	QT	Y	12 months
Funato et al., 1982 [24]	1	Y	TR	N	QT and R	Y	18 months
Kudo et al., 1982 [25]	1	Y	N	N	N	N	-
Scheibel et al., 1982 [26]	1	Y	TR	N	QT and R	Y	30 months
Yagihashi et al., 1982 [27]	1	Y	N	N	N	N	-
Yamashiro et al., 1982 [28]	1	Y	TR	N	N	N/A	N/A
Booth et al., 1983 [29]	1	Y	TR	N	N	Y	24 months
El Shafie et al., 1983 [9]	2	2Y	1 TR, 1 PR	2N	1N, 1 QT and R	1Y, 1N/A	36 months, N/A
Granot et al., 1983 [30]	1	Y	TR	N	N	Y	11 months
Bunnett et al., 1984 [31]	1	Y	TR	N	N/A	N/A	N/A
Schuman et al., 1984 [32]	1	Y	TR	N	N	Y	18 days
Socha et al., 1984 [14]	2	1N, 1Y	1 TR, 1N/A	1N, 1N/A	2N	1N, 1Y	24 months, N/A
Dorney et al; 1984 [33]	1	Y	TR	N	N	Y	12 months
Oberg et al., 1986 [13]	1	Y (2x)	PR	Y	N	N/A	N/A
Quak et al., 1988 [34]	1	Y	TR	N	N	Y	6 months
Lacey et al., 1989 [35]	1	Y	PR	N	RP (2x) + R + QT	Y	72 months
Davies et al., 1990 [36]	1	Y	TR	N	N	N/A	N/A
Kimura et al., 1993 [37]	3	3Y	3 TR	3 N	3 N/A	3 N/A	3 N/A
Albers et al., 1998 [38]	1	Y	TR	N	N	N/A	N/A
Murphy et al., 2000 [39]	6	6Y	6 TR	6N	6N	6Y	5 to 14 years
Rodriguez et al., 2000 [40]	1	Y	TR	N	N	Y	24 months
Riebel et al., 2002 [41]	1	Y	TR	N	N	Y	N/A
Wildhaber et al., 2003 [42]	1	Y	TR	N	R + QT	Y	24 months
Bourgois et. al., 2004 [43]	1	Y	TR	N	N	Y	9 months
Gesundheit et al., 2004 [44]	1	Y	TR	N	N	Y	27 months
Reindl et al., 2004 [45]	2	2Y	2 TR	2N	2N	2 N/A	2 N/A
Zhang et al., 2008 [46]	1	Y	TR	N	N	Y	8 months
Bourdeaut et al., 2009 [7]	19	19 Y	17 TR, 2 PR	18 N, 1Y	12QT, 7N	17Y, 2N	17 N/A, 5 Stable disease, 2 progressive disease
LeLeiko et al., 2010 [47]	1	Y	TR	N	N	Y	N/A
Husain et al., 2011 [10]	1	Y	TR	Y	N	Y	N/A
Kargl et al., 2013 [48]	1	Y	TR	N	N	Y	N/A
Kanik et al., 2014 [11]	1	Y	TR	N	N	Y	12 months
Toro, Gonzales et al., 2014 [49]	1	Y	TR	N	N	Y	40 months
Kumar et al., 2015 [50]	1	N	N	N	QT	N/A	N/A
Minson et al., 2015 [51]	1	Y	PR	O	QT	N	18 months
Whitfield et al., 2015 [52]	1	N	N	O	QT	N/A	N/A
Romanyshyn et al., 2017 [53]	1	Y	TR	N	N	Y	Remission
Sintusek et al., 2017 [54]	1	Y	TR	N	QT	Y	36 months
Tanzi et al., 2017 [55]	1	Y	TR	N	QT	Y	3 months
Czkwianianc et al., 2018 [56]	2	2Y	2 TR	2N	1QT and R, 1QT	2Y	24-30 months
Kabalan et al., 2018 [57]	1	Y	PR	N	QT	Y	Stable disease for 84 months
Sugita et al., 2020 [58]	1	Y	TR	O	N	Y	12 months
Uskova et al., 2020 [59]	6	6Y	4 TR, 2 PR	6N	5QT, 1N	5Y 1N	1N/A, 5-18 months, 2 under treatment
Yeh et al., 2020 [60]	1	Y	PR	N	N	Y	18 days
Liu et al, 2021 [61]	2	2Y	2 N/A	2O	2QT	2Y	2 remission
Shahid et al., 2021 [62]	7	3Y, 4N/A	2 TR, 5N/A	2O, 5N	7QT	1N, 1Y, 5N/A	5N/A, 1 death 13 months later, 1 survival 40 months

Concerning survival, 68 (70%) out of 96 patients were described as alive after treatment, with follow-up times varying greatly between reports, ranging from two weeks to 14 years. Nine out of 96 (9.3%) patients were described as dead: eight due to the disease or treatment complications, and one due to other causes unrelated to the tumor or treatment. One patient was diagnosed with a neuroblastic tumor during an autopsy. Recurrence was described in only three (3.1%) of 96 cases, all of whom achieved remission after therapy and overall remission was achieved for 53 (55%) out of 96 patients. Survival information was incomplete in the remaining 19 (19.7%) out of 96 patients.

Discussion

Serum VIP was first isolated by Sami Said and Viktor Mutt in 1970, respectively, an Egyptian and an Estonian, who collaborated in a Swedish laboratory for the discovery [63]. This peptide is similar in structure to glucagon, secretin, and gastric inhibitory peptides and induces a myriad of effects. At metabolic levels, it stimulates lipolysis and glycogenolysis and is inotropic. In the stomach, it has antihistaminic effects and stimulates acid secretion. Serum VIP also stimulates the secretion of alkaline pancreatic juices. In the small intestine, however, it is a potent stimulator of electrolyte and water secretion and adenylate cyclase production, which explains the pathophysiology of refractory diarrhea in patients with VIP-producing neuroblastic tumors [16].

In this review (86.5% of cases), diarrhea precedes the diagnosis of the tumor, and the patient remains a long time (an average of eight months) without adequate treatment, impacting the child's general condition and enhancing the risk of the tumor spreading, as can be seen in the photograph reproduced by Kanik et al. (2014) [11]. On the other hand, the occurrence of watery diarrhea during NB treatment, in 13.5% of cases, suggests to the experienced oncologist that the tumor may be differentiating.

Serum VIP production in neuroblastic tumors seems to occur during tumor differentiation. This could justify the better prognosis of patients with this paraneoplastic syndrome, since it would indicate evolution from the immature and aggressive form of the neuroblastic tumor to the more differentiated histological type [44], provided that these patients have access to the necessary support measures to control diarrhea, dehydration, and metabolic and acid-base disturbances caused by the excretion of VIP.

The MYCN proto-oncogene was amplified in 7.2% (7/96) of NB cases with watery diarrhea. MYCN is a genetic marker found in around 20% to 30% of NB. This proto-oncogene is located on chromosome 2, and its amplification correlates with aggressive, metastatic, and chemotherapy-refractory tumors. It is rare in VIP-secreting neuroblastic tumors since this peptide is generally associated with differentiated tumors (ganglioneuroma) or tumors undergoing differentiation (ganglioneuroblastoma) [64].

The diarrhea described in most patients with VIP-producing neuroblastic tumors has a watery appearance, and varying frequencies, but is multiple throughout the day, if not continuous. Commonly, 62.5% (60/96) of cases are associated with hydroelectrolytic or acid-base disorders. Hypokalemia and metabolic acidosis were frequent, affecting 59 (98.3%) of 60 patients and 23 (38.9%) of 59 patients, respectively. The association between metabolic disorders and multiple potentially serious hydroelectrolytic disorders (hypokalemia, hyponatremia, hypophosphatemia, hypochloremia) requires special attention from the pediatrician in the control and therapeutic planning of these patients.

Despite the watery description present in most articles, our knowledge of the action of VIP in the small intestine allows us to understand that these patients have diarrhea with a secretory mechanism. In view of the main etiologies of chronic secretory diarrhea, as explained in Rodriguez et al. [40], it is apparent that most derive from congenital causes; two are bacterial causes, one of an immunological cause, and one of a neoplastic cause. Considering all etiologies, neural crest tumors are the curable cause with the greatest impact on the patient's quality of life in the event of a delayed diagnosis, due to the possible evolution of the tumor into metastases. As such, in the case of chronic diarrhea of unclear etiology, it is important to investigate the hypothesis of neural crest tumors.

The diagnosis of neuroblastic tumors is based on anamnesis, physical examination, laboratory tests, and imaging tests (Table 3). Neuroblastic tumors mainly affect infants and preschoolers and are very rare after five years of age. During anamnesis, in addition to the characteristics of diarrhea, it is important to investigate signs and symptoms of NB, such as fever, bone pain, and adenomegaly. During physical examination, it is vital to examine blood pressure control, and carefully evaluate the presence of palpable tumors in the abdomen or paravertebral region, enlarged lymph nodes, and periorbicular ecchymosis [5]. Small neuroblastic tumors located in the mediastinum or abdomen (15%) are generally asymptomatic and are diagnosed through imaging tests. Considering that the main location of neuroblastic tumors is in the abdomen (71.8% of the cases in our review), an ultrasound performed by an experienced professional may help with diagnosis. Chest X-rays to check for mediastinal tumors should also be ordered.

**Table 3:** Suggested investigation of neural crest tumors

Examination aspect		Relevant aspects					
Anamnesis		Persistent watery diarrhea, hydroelectrolytic and metabolic disorders, failure to thrive, abdominal pain, bone pain, fever					
Physical examination		Hypertension, palpable abdominal mass, adenomegaly					
Laboratory tests		Measurement of urinary catecholamines, serum electrolytes, serum vasoactive intestinal peptide					
Imaging tests		Chest X-ray, abdominal ultrasound, MIBG whole body imaging, myelogram, and bone marrow biopsy					

Regarding laboratory tests, dosing of the catecholamines vanillyl mandelic acid (VMA) and homovanillic acid (HVA), as well as the measuring VIP, can clarify the diagnosis. Figure 3 illustrates how VIP levels related to this paraneoplastic syndrome are mostly concentrated at levels up to 20 x ULN but are not limited to this range, given the reports of cases with up to 188 times the ULN and also cases with levels under the ULN that are reduced after surgical removal of the tumor. The search for malignant cells in the bone marrow with a myelogram and biopsy can also be requested if a metastatic tumor is suspected. Whole-body imaging with radioactive iodine 131 or 123 (MIBG) is the "gold standard" test for patients with neuroblastoma or other neuroblastic tumors, has sensitivity and specificity of 90% and 100%, respectively, and is performed for primary tumor investigation, staging, and follow-up [65]. Obviously, in cases of suspected VIP hypersecretion syndrome, a specialist in pediatric oncology should be consulted to advise on tests and treatment.

The treatment of diarrhea secondary to a VIP-producing neuroblastic tumor has two main objectives: keeping the patient stable by controlling hydroelectrolytic and metabolic disorders and tumor resection. Diarrhea in VIP-producing tumors can be alleviated by the administration of somatostatin and its analogs. Somatostatin is a hormone secreted by hypothalamic neurons and is considered to regulate endocrine secretion [42]. Octreotide, used by several authors in our review, is a peptide that pharmacologically simulates natural somatostatin, is a more potent inhibitor of growth hormone, glucagon, and insulin than the natural hormone, and is used clinically to relieve symptoms of various gastroenteropancreatic endocrine tumors [10]. Regardless, surgery to remove the VIP-producing tumor is the definitive form of treatment. When the tumor is localized, total resection of the tumor, which was possible in 72 (75%) of 96 of the cases in our series, is curative and controls diarrhea. In unresectable or metastatic tumors, partial surgery, radiotherapy, and chemotherapy can be used. Therefore, metastatic and unresectable tumors are poor prognostic factors for VIP-producing neuroblastic tumors. Most authors did not describe follow-up periods above 40 months; hence, survival rates were not assessed in this review.

## Conclusions

We carried out an epidemiological study of children with VIP-producing neural crest tumors in childhood. The patients were mostly under five years of age, and most had no diagnosis of the tumor at the onset of diarrhea. Hypokalemia was the most frequent disorder, and it was generally associated with dehydration, hyponatremia, and metabolic acidosis. Health professionals should consider the hypothesis of diarrhea secondary to VIP-producing tumors in children with difficult-to-control secretory diarrhea without a defined etiology in the usual investigations. Correct and early diagnosis of the etiology of diarrhea drastically changes the prognosis and quality of life of these patients.
